# Phylogenetic and epidemiological insights into centenarians’ resilience to COVID-19: exploring the role of past coronavirus pandemics

**DOI:** 10.3389/fmicb.2025.1572763

**Published:** 2025-04-17

**Authors:** Greta Romano, Alessandro Ferrari, Fausto Baldanti

**Affiliations:** ^1^Department of Microbiology and Virology, Fondazione IRCCS Policlinico San Matteo, Pavia, Italy; ^2^National PhD Programme in One Health Approaches to Infectious Diseases and Life Science Research, Department of Public Health, Experimental and Forensic Medicine, University of Pavia, Pavia, Italy; ^3^Department of Clinical, Surgical, Diagnostic and Paediatric Sciences, University of Pavia, Pavia, Italy

**Keywords:** centenarians, COVID-19, immunity, betacoronaviridae, HCoV-OC43, historical mortality, pandemics, cross-protection

## Abstract

In the 20th and 21st centuries, humanity has faced several global crises, including world wars, the 1918 Spanish flu, and the recent COVID-19 pandemic. Notably, the COVID-19 pandemic caused significant mortality, particularly among older adults, while younger ages were less affected. Strikingly, according to the Italian National Institute of Statistics (Istat), centenarians (aged 100 and above) in Italy experienced no significant increase in mortality in 2020. This retrospective study hypothesizes that elderly people may have developed an immune response that offered protection against COVID-19, potentially linked to their exposure to a specific past infectious event. We examined historical mortality data from 1872 to 2021 and performed phylogenetics analysis on sequencing data to explore the possibility that centenarians may have encountered another Coronavirus (misidentified as Russian Flu), which could have contributed to their resilience. This research provides insights into the adaptive responses of the most vulnerable populations, symbolically comparing them to the “left-standing trees” following catastrophic events.

## 1 Introduction

Over the centuries, many world events have plagued the population, among which should be mentioned the World Wars (I and II) and a series of pandemics caused by infectious agents such as the Spanish flu or SARS-Cov-2. The most recent COVID-19 pandemic has been a particular impact event because of its high infectiousness and mortality. The report jointly produced by the Italian National Institute of Statistics (ISTAT) and the Italian Higher Institute of Health (ISS) presents a summary of the main characteristics of the spread of COVID-19 and its impact on total mortality over the two-year period 2020–2021 (ISTAT, 2020). The deaths associated with the diagnosis of SARS-CoV-2 infection were 145,334 as reported by the Integrated COVID-19 National Surveillance System of the ISS, and they occurred by January 31, 2022 (ISTAT, 2021). Among the age groups, the most significant contribution to the excess of deaths was due to the increase in deaths in the population aged 65 years and over. However, unlike the other age groups of the elderly population, those who reached or exceeded 105 years of age experienced no significant increase in deaths during 2019 compared to 2020, the first year of the COVID-19 pandemic when no specific vaccine was available (ISTAT, 2019; ISTAT, 2020). The hypothesis that we support in this study is that today’s centenarians could already have an immunological response to the COVID-19 virus. Through the analysis of mortality historical data and COVID-19 spread studies, it can be suggested that centenarians may have encountered a progenitor of SARS-Cov-2 that circulated in the early 1900s. Thus, this study analyzes Italian mortality data in the period 1872 to 2021 integrating viral phylogeny studies to shed light on the real possibility that the most fragile age group of our population is actually the *left-standing tree* of our era.

## 2 Materials and methods

All the data presented in this article were downloaded from three different sources: the Italian National Institute of Statistics (ISTAT) (https://www.istat.it/), the Italian Higher Institute of Health (ISS) (https://www.iss.it/) and the Human Mortality Database (HMD) (https://www.mortality.org/). HMD data could be estimates (of population size or numbers of deaths), not actual counts and therefore may be expressed as non-integers.

The maximum likelihood (ML) phylogenetic tree of the SARS-CoV-2 dataset was constructed using IQ-TREE multicore version 2.3.3 (Minh et al., 2020) under the GTR + G + I nucleotide substitution model selected according to BIC score (Bayesian Information Criterion), as it was the best-fitting model selected by ModelFinder (Kalyaanamoorthy et al., 2017). The robustness of the branches was evaluated using the Shimodaira–Hasegawa approximate likelihood-ratio test (Shimodaira and Hasegawa, 1999) and the ultrafast bootstrap approximation tests (Hoang et al., 2017).

## 3 Results

Following the emergence of the new Severe Acute Respiratory Syndrome Coronavirus 2 (SARS-CoV-2), the infection known as novel Coronavirus disease 2019 (COVID-19) has impacted all countries and resulted in an excess mortality rate globally (World Health Organization, 2021). Coronaviruses, single positive-stranded RNA viruses belonging to the Coronaviridae family, are found in various animal species and comprise the genera *Alpha*, *Beta*, *Delta*, and *Gammacoronavirus*. Six human CoV types, comprising two alpha and four beta CoVs, have been identified to cause respiratory illnesses in humans, among which are SARS-CoV and MERS-CoV.

**Figure 1:**
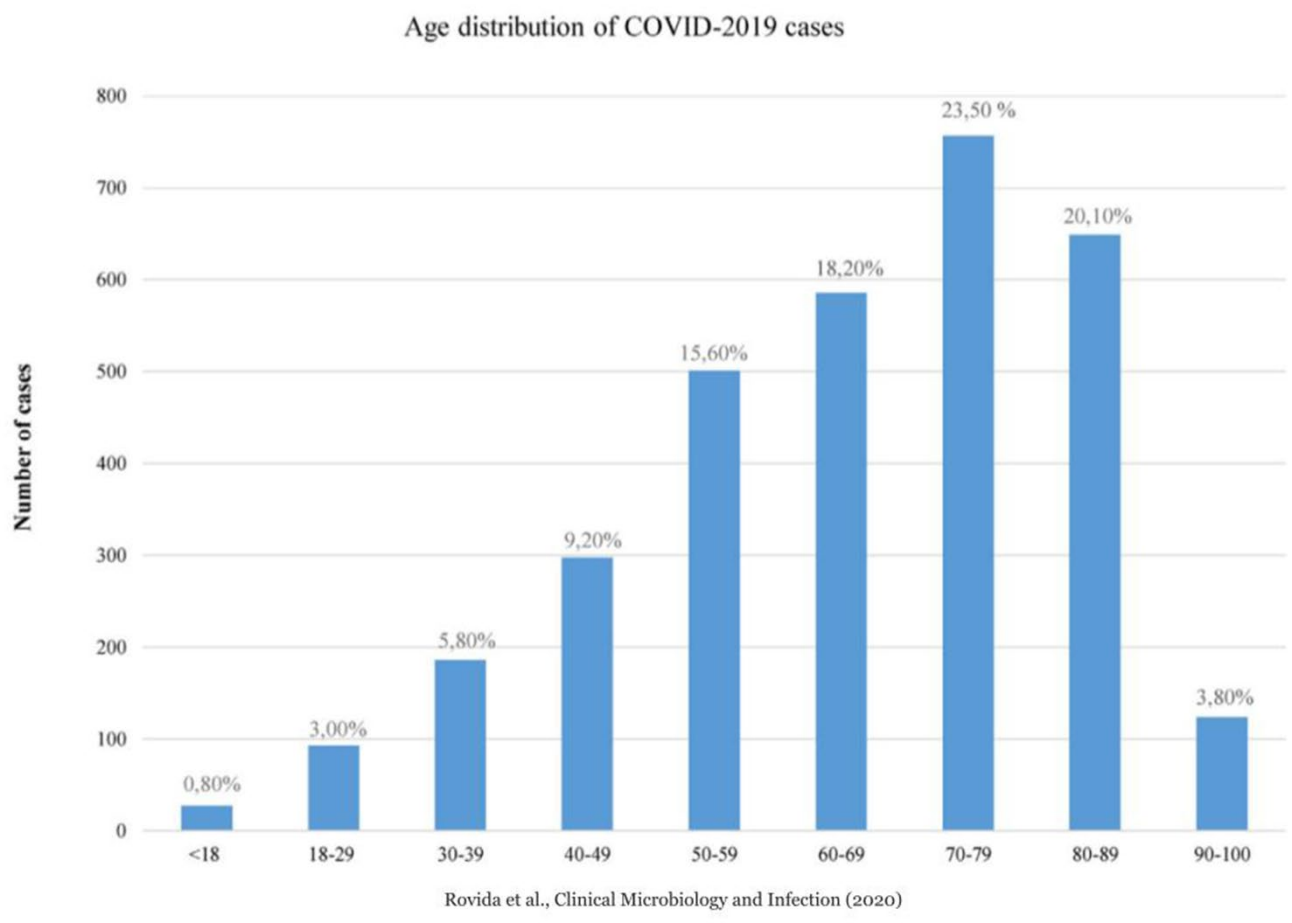
Age distribution of COVID-2019 cases, Lombardy Region, Italy 2020 (Courtesy of *Rovida et al. Clin Microbiol Infect. 2020*).

**Figure 2:**
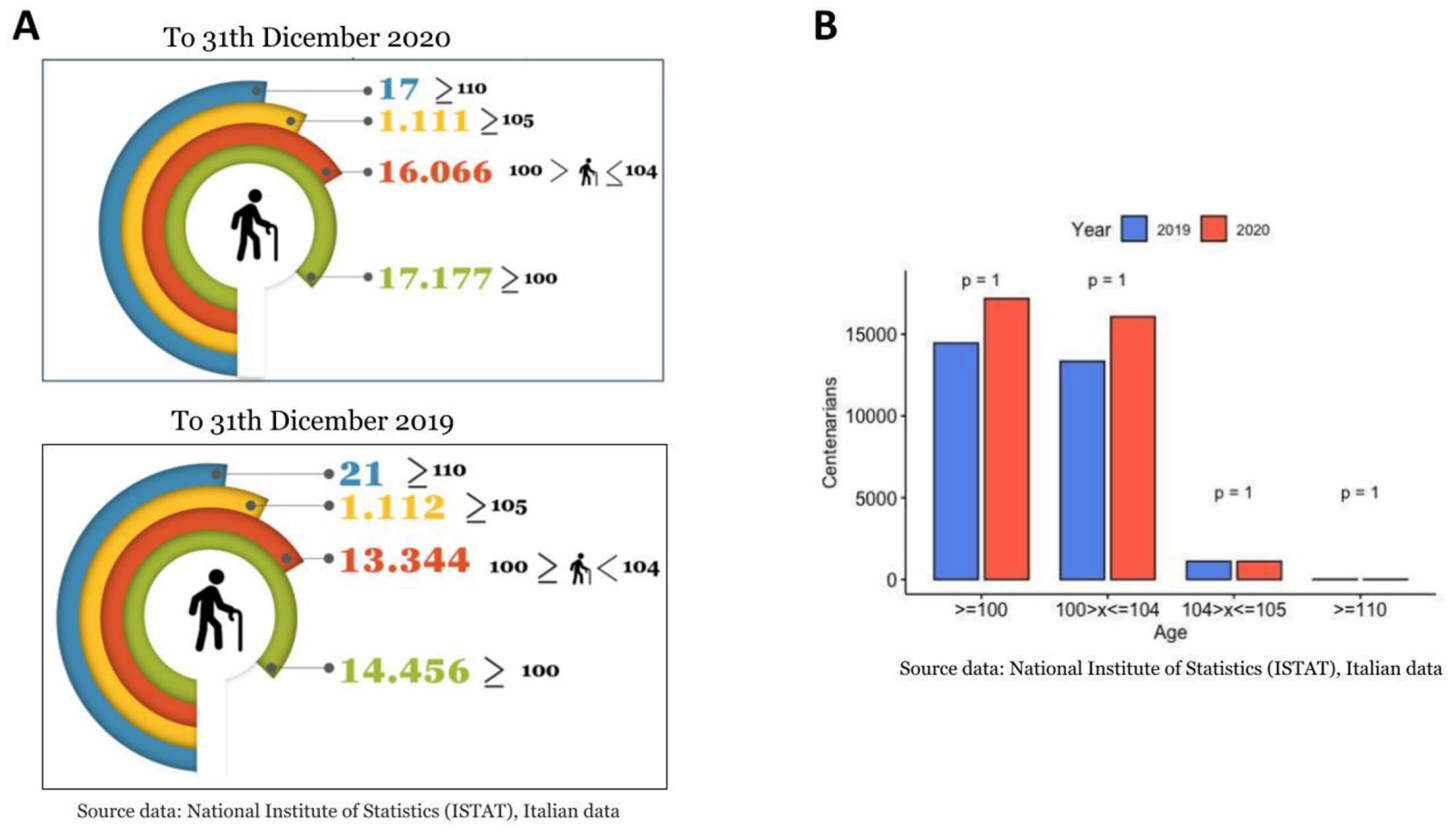
Centenarians in Italy. **(A)** Number of centenarians according to ISTAT in 2020 compared with the 2019, grouped by age. **(B)** Statistical comparison (*t-test method*) of centenarians number between two years.

At the beginning of 2020, the rapid spread of the SARS-Cov-2 virus had a catastrophic effect worldwide, resulting in more than 6 million deaths. COVID-19 has emerged as the most significant global health crisis since the beginning of the 1918 influenza pandemic (ISTAT, 2020). In 2020, 746.324 people died in Italy; 108.496 more cases than the 2015–2019 average. There were 227.350 deaths from circulatory diseases, 177.858 from cancer, 78.673 from COVID-19, and 57.113 from respiratory diseases (ISTAT, 2019). According to the Italian Superior Institute of Health (ISS) (ISTAT, 2019), more than a quarter of the deaths occurred in Lombardy (Northern Italy), the first western region impacted by the pandemic, where 30.341 people have died since the start of the pandemic, accounting for 28.4% of the total number of deaths from COVID in Italy, with a median age of 81 (https://www.iss.it/). Moreover, Rovida et al. (2020) reported that among a total of 3,220 SARS-CoV-2-positive patients in Lombardy, collected at the Regional Reference Laboratory (Molecular Virology Unit, Fondazione IRCCS Policlinico San Matteo Pavia, Italy Lombardy, Italy), only 0.80% (27/3220) were pediatric (<18 years), while 99.2% were adult (>18 years) (Figure 1). In addition, the COVID-19 cases included in the 90–100 age group were numerically smaller than those included in the other age groups (Figure 1) (Rovida et al., 2020). The regional overview of Lombardy was then verified and confirmed for the entire country by ISTAT (2021, 2019). As of January 01, 2021, there were 17,177 persons over 100 years of age in Italy. Of these, 16.066 were between 100 and 104, 1,011 were over 105 years old, and 17 were over 110 years old (Figure 2, Panel A) (ISTAT, 2021). Comparing these data with the number of centenarians present on 1 January 2020: 14456 were over 100 years of age, of which 13.344 were between 100 and 104, 1,112 were over 105, and 21 were over 110 (Figure 2, Panel A) (ISTAT, 2019). Thus, unlike the other age groups of the elderly population, those who reached or exceeded 105 years of age did not experience significant death growth during 2020, the first year of the COVID-19 pandemic (Figure 2, Panel B) (ISTAT, 2021; ISTAT, 2019). The mortality rate (excess mortality rate) increased with age. Thus, one would expect the latter decades, the most frail, to have a higher risk of death. It would seem, however, that COVID-19 was an exception.

At the beginning of the COVID-19 pandemic, several authors searched for the roots of this virus and possible ancestors among different members of the *Coronaviridae* family. Some of this research suggests that the so-called “Russian Influenza” or “Russian Flu” pandemic (1889–1895) shares several characteristics with COVID-19 (Honigsbaum and Krishnan, 2020; Brüssow and Brüssow, 2021). “Russian Flu” was a deadly pandemic (1889–1895) that killed an estimated 1 million people worldwide. It started in Bukhara (modern Uzbekistan) in 1889 and spread all over Europe through Russian traffic and rail lines (Supplementary Figure 1). The epidemic was named “Russian Flu” because the causative agent could only be hypothesized (Influenza A) due to a seroarchaeological approach (i.e., the detection of antibodies to influenza infection in the sera of elderly people) to indirectly reconstruct influenza epidemics (Altschuler et al., 2009).

The age-specific mortality rate for influenza is traditionally U-shaped, with high mortality rates among infants, young children, and the elderly, whereas mortality remains very low across all ages. However, a distinct pattern emerged for Spanish Flu pandemics that occurred between 1918 and 1920. The mortality pattern of this pandemic event was characterized by a W-shaped curve, which included a third mortality peak observed among individuals aged 18–30 years (Morens et al., 2021; Ahmed et al., 2007). Consequently, several authors considered that individuals over 30 years old may have had some immunity to the 1918 influenza pandemic strain, which was not present in the younger adult population (18–30 year olds) (Morens et al., 2021; Ahmed et al., 2007). The principle hypothesis regards people born before 1889 (>30 years old in 1918) who were likely to have been exposed to H1-type influenza viruses circulating before 1889 (Ahmed et al., 2007). In comparison, people born in or after 1889 would have been immunologically naive to the 1918 H1 pandemic strain (that is, at least to the HA of the 1918 H1 strain) (Ahmed et al., 2007). However, the factor of World War I must also be considered in this dissertation. Although the age range of Italian soldiers called to arms during the First World War was between 18 and 44, the battle conditions (stress, fatigue, chemical exposure) may have weakened the soldiers’ immune systems, thereby increasing their vulnerability to disease. Finally, a further factor to consider is the absence of antibiotic drugs that became available after 1940. Thus, a weakened immune system may have been the consequence of bacterial superinfections.

However, similar mortality rates were observed in young men and women who were not involved in the war (Ahmed et al., 2007). This can be explained by the low socioeconomic and health status of the Italian population in 1918 due to the war economy, heavy working conditions, unemployment, and social crisis.

**Figure 3:**
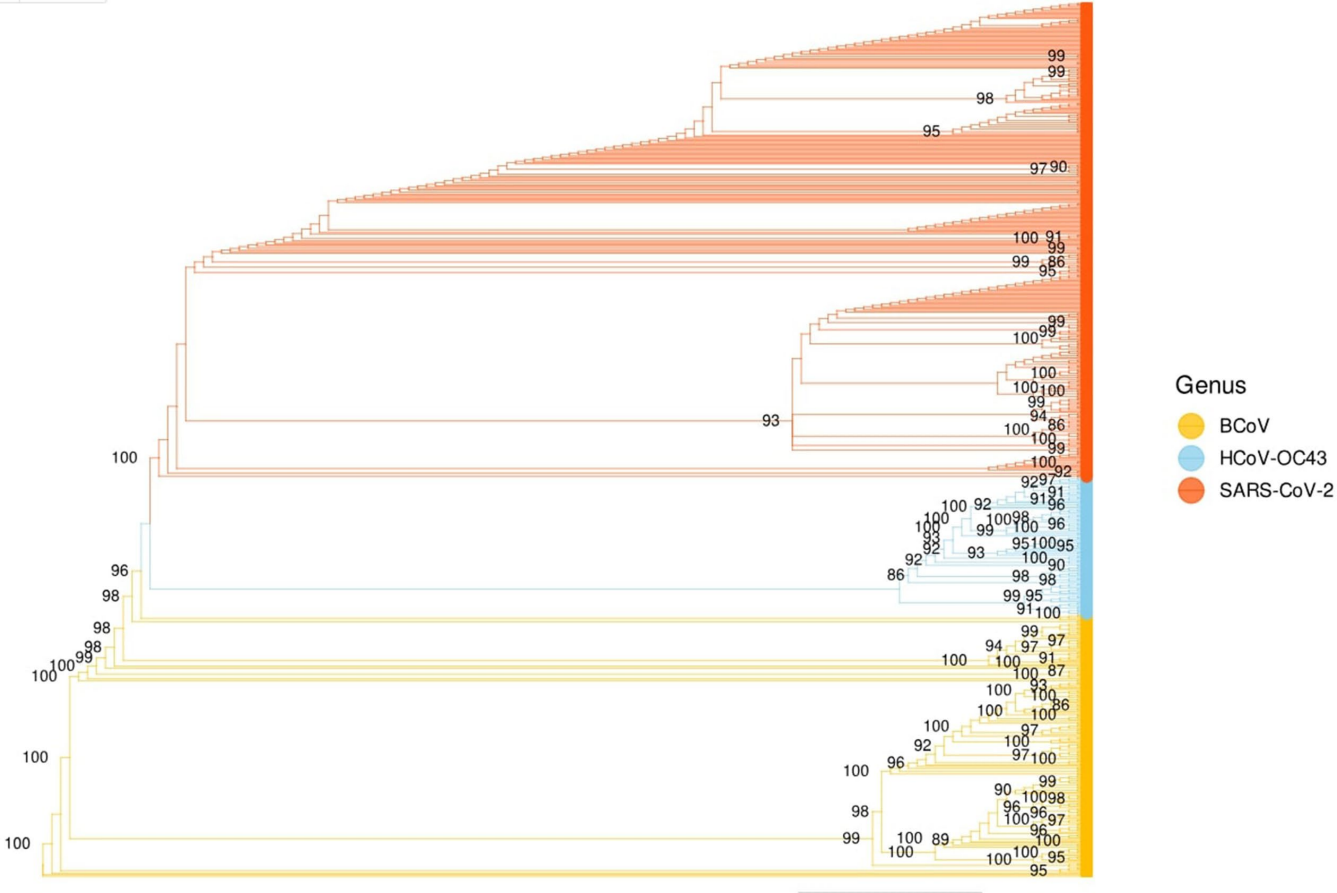
Maximum Likelihood (ML) phylogenetic tree. The ML tree contains 346 SARS-CoV-2 Lombardy strains (Alteri et al., 2021), 100 HCoV-OC43 and 191 Bovine Coronavirus strains downloaded from GenBank.

The Russian flu pandemic was well reported in chronicles of the time published in the British Medical Journal (Kousoulis and Tsoucalas, 2017), as well as press dispatch (Honigsbaum, 2010). However, some clinical characteristics of the “Russian Flu” are closer to COVID-19 than classical influenza. Multiorgan symptoms ranging from the respiratory system (dry spasmodic cough, bronchitis, pneumonia) to gastrointestinal symptoms (nausea, vomiting, diarrhea) to marked neurological symptoms were observed (Honigsbaum, 2010). The Russian flu pandemic discussion took an unexpected turn when the sequence of human *Betacoronavirus* OC43 (HCoV-OC43) was determined (Brüssow and Brüssow, 2021; Vijgen et al., 2005). *Betacoronaviruses* comprises HCoV-OC43 (Embecovirus Lineage A), and SARS-CoV and SARS-CoV-2 (Sarbecovirus Lineage B). HCoV-OC43 causes mild upper respiratory tract infections and rarely pneumonia in neonates and older people with underlying illnesses. Moreover, HCoV-OC43 shared 93.5% nucleotide sequence identity with the S (spike) gene and 98% with the E (minor envelope) gene of bovine Coronavirus (BCoV) genome (Vijgen et al., 2005). BCoV was shown to be the closest relative of HCoV-OC43 suggesting a potential recombination event with HCoV-OC43 (Vijgen et al., 2005). In addition, three evolutionary models estimated the time to the most recent common ancestor of HCoV-OC43 and BCoV as 1891, 1873, and 1890, respectively, with an annual substitution rate of 4.3 × 10^−4^ (Vijgen et al., 2005). Thus, several authors support the idea that HCoV-OC43 might be the ideal candidate for the “Russian Flu agent” for several reasons: age range, symptomatology, and possible derivation from a bovine Coronavirus (spillover), whose common ancestor was in the late 1800s (Honigsbaum and Krishnan, 2020; Brüssow and Brüssow, 2021; Vijgen et al., 2005). To confirm this hypothesis, complete genome analyses of SARS-Cov-2 were performed in comparison to the HCoV-OC43 and BCoV genomes. We performed the phylogenetic analysis using data published in Alteri et al. (2021) related to first-wave SARS-CoV-2 sequences in Lombardy (Alteri et al., 2021). In this study (Alteri et al., 2021), whole-genome sequencing was performed for 346 SARS-CoV-2 strains obtained from individuals from various geographical areas (Lombardy, Italy) in a time span of 2 months (Alteri et al., 2021). The dataset was integrated using 100 sequences of human Coronavirus OC43 and 191 bovine Coronavirus sequences (GenBank). A Maximum Likelihood phylogenetic tree of SARS-CoV-2, HCoV-OC43, and BCoV was constructed based on whole-genome sequence alignment. The SARS-CoV forms a separate branch, although there is strong support for the monophyly of SARS-CoV with the group 2 Coronaviruses, HCoV-OC43 and BCoV (Figure 3).

**Figure 4:**
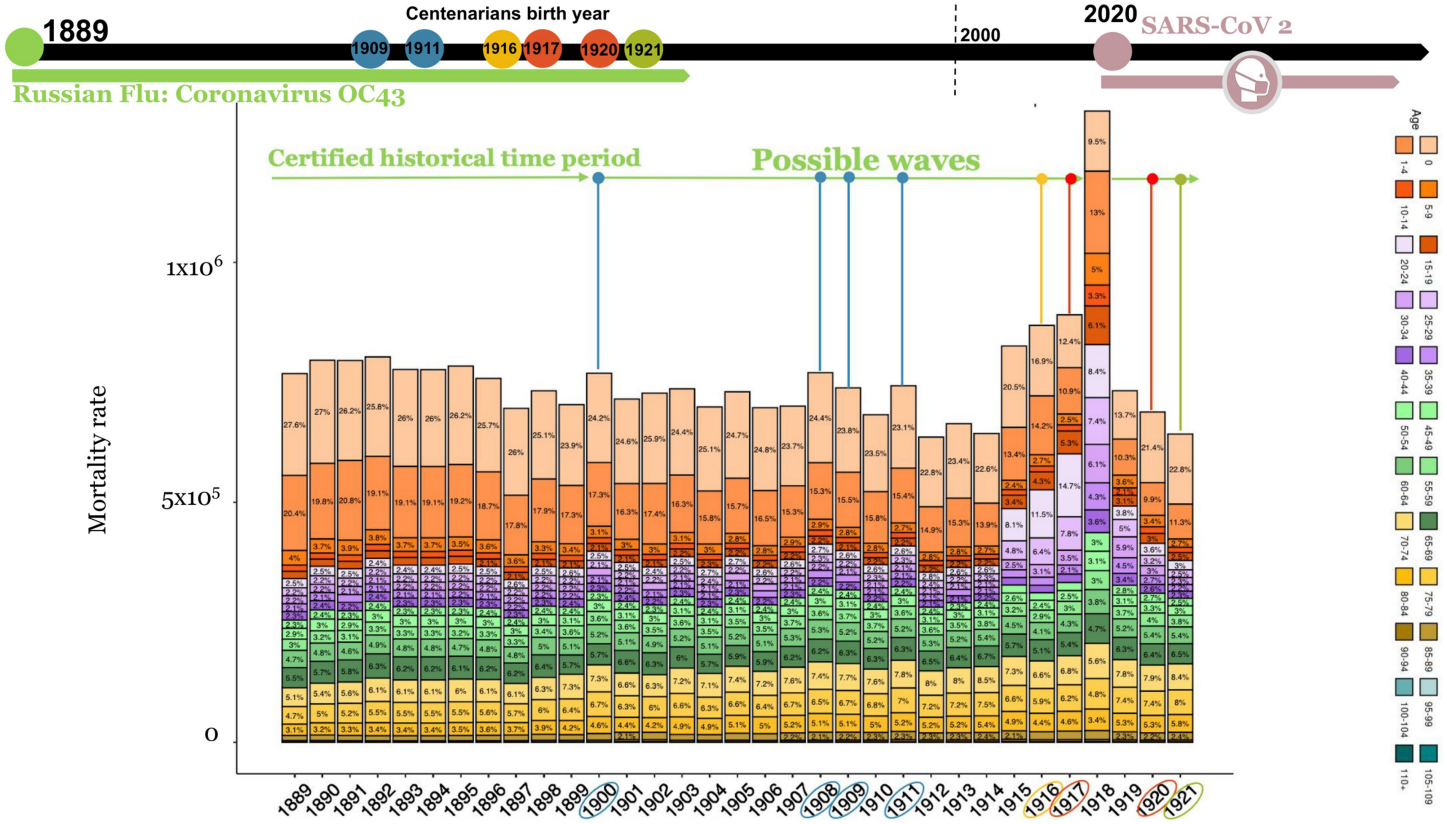
Italian deaths rate from 1889 to 1921 divided for 24 age groups. Possible wave lines are colored based on centenarian’s birth years (circles in timeline). Data was recovered by HMD source and processed using R studio. Since some HMD data could be estimated they are expressed as non-integers.

Because the “Russian Flu” arrived in Italy in late 1889 (Supplementary Figure 1) and no specific vaccines were available at the time, this pandemic presumably spread in successive waves over the years (Figure 4: 1889–1895, 1900, 1908, 1911), where it can be observed a mortality excess rate (especially for the 0–10 years and 45–69 years age groups). Thus, it is hypothesized that in the absence of a vaccine, the excess mortality peaks could be subsequent to waves of Russian Flu caused by HCoV-OC43. The adults and children who survived the “Russian Flu” may have developed robust immunity. In chronological order, the first group to develop protective immunity should have been the present-day centenarians who were children at the time. Indeed, centenarians that were alive in 2021 were presumably born between 1909 (the oldest) and 1921 (the youngest). The last recorded peak of high mortality, presumably due to the Russian flu, ranged from 1908 to 1911 (Figure 4).

The SARS-CoV-2 pandemic of 2020 had, as a main consequence, an increase in the number of studies regarding prior immunity. Interesting findings have been obtained from research on anti-S-neutralizing antibodies. In particular, both Italian (Trombetta et al., 2024) and American (Foley et al., 2021) research on centenarians living in long-term care facilities has shown that the elderly immune system is still capable of generating an antibody response to SARS-CoV-2 infection and that the produced antibodies have neutralizing capabilities.

Besides B-cell immunity, several research groups have sought to improve the knowledge of T-cell immunity to understand whether it may play a role in protecting against viral infections of unknown etiologies. Some of these studies showed how SARS-CoV-2-reactive T cells are present in some healthy individuals who have been unexposed to the virus (Cassaniti et al., 2021; Dos Santos Alves et al., 2024). Although the anti-SARS-CoV-2 antibody response primarily targets variants of S proteins, T cells contribute to immunity by identifying conserved regions across multiple SARS-CoV-2 proteins (Dos Santos Alves et al., 2024). However, the origin of SARS-CoV-2 cross-reactive T cells in unexposed humans is unclear. Cassaniti et al. showed that the cross-reactive SARS-CoV-2 T-cell response in uninfected patients may be due to previous infections with other common Coronaviruses (Cassaniti et al., 2021). The findings of these studies suggest that the long-term SARS-CoV-2 T-cell response might last in spite of a waning humoral response (Cassaniti et al., 2021). In addition, these T cells could be elicited by the common cold Coronavirus OC43, as shown in the manuscript of Dos Santos Alves et al. (2024). The authors exploited Human leukocyte antigen (HLA) transgenic mouse models to perform sequential infections with human Coronavirus OC43 and SARS-CoV-2. The results show that OC43 elicits cross-protective immunity against SARS-CoV-2, which is partially dependent on CD4 + T cells (Dos Santos Alves et al., 2024).

In addition, Mbogori et al. (2024) identified genomic similarities between endemic coronaviruses and SARS-CoV-2, indicating that immune priming from past coronavirus exposures could contribute to protection against novel strains. These findings align with those of previous studies emphasizing the role of cross-reactive T-cell immunity in mitigating severe COVID-19 outcomes (Mbogori et al., 2024).

The hypothesis discussed in this manuscript has also been suggested for other viruses, including bird Flu (Kozlov, 2024). Indeed, according to Kozlov (2024), centenarians might have more protection than younger populations because they were probably exposed to ancestor’s ‘matched’ strains (i.e., viral structures conserved over time) during childhood. This manuscript is not intended to provide an undisputed proof but is offered as an indirect analysis of actual historical events. The conclusion we refer to the reader as to why Italian centenarians survived the pandemic is the result of a careful study of mortality in Italy obtained through established sources.

## 4 Conclusion

Research into the origins of COVID-19 has prompted several researchers to examine not only current Coronaviruses but also past pandemics, such as the “Russian Flu” of 1889–1895, for possible ancestors or correlations with SARS-CoV-2 (Honigsbaum and Krishnan, 2020; Brüssow and Brüssow, 2021; Vijgen et al., 2005). Although the Russian Flu infectious agent was initially hypothesized to be an influenza virus, some researchers now suggest that it may actually have been a Coronavirus, the human beta-Coronavirus HCoV-OC43, belonging to the same family as SARS-CoV and SARS-CoV-2 (Honigsbaum and Krishnan, 2020; Brüssow and Brüssow, 2021; Vijgen et al., 2005). HCoV-OC43, which causes mostly mild respiratory infections today, bears significant genetic similarity to bovine Coronavirus (BCoV), suggesting that it may have originated from a spillover event from cattle to humans in the late 19th century (Vijgen et al., 2005). Molecular and phylogenetic studies have estimated that the last common ancestor of HCoV-OC43 and BCoV dates from 1873 to 1891, a period compatible with the emergence of the Russian influenza pandemic (Vijgen et al., 2005). The phylogenetic analysis performed using data from Alteri et al. (2021) on first-wave SARS-CoV-2 sequences in Lombardy showed strong support for the monophyly of SARS-CoV-2 with HCoV-OC43 and BCoV sequences.

The clinical features of Russian influenza strengthen the suspicion of similarity with COVID-19, rather than seasonal influenza. In fact, during the epidemic, in addition to common respiratory symptoms such as bronchitis and dry asthma, many patients presented with gastrointestinal (nausea, vomiting, diarrhea) and neurological symptoms, reminiscent of the broader systemic manifestations observed in patients with COVID-19 (Brüssow and Brüssow, 2021). Another significant aspect concerns B and T cell immunity, which has been extensively studied in recent years to understand the immune response to SARS-CoV-2 (Trombetta et al., 2024; Foley et al., 2021; Cassaniti et al., 2021; Dos Santos Alves et al., 2024; Mbogori et al., 2024; Stanley et al., 2024). Regarding centenarians, some studies have demonstrated how the immune system of centenarians generates anti-S-neutralizing antibodies with neutralizing capabilities in response to SARS-CoV-2 infection (Trombetta et al., 2024; Foley et al., 2021).

Some individuals not exposed to SARS-CoV-2 still have reactive T cells, and research has shown that these responses could result from previous infections with common Coronaviruses, such as HCoV-OC43 (Cassaniti et al., 2021; Dos Santos Alves et al., 2024). Moreover, Mbogori et al. (2024) also pointed out that there are shared epitopes between HCoV-OC43 and SARS-CoV-2 viruses, which supports the idea that a long-lasting T-cell immunity mechanism is possible (Mbogori et al., 2024). Stanley et al. discovered in 2024 that SARS-CoV-2 shares limited antibody protection with endemic coronaviruses, yet the long-lasting immunity provided by T-cell immunity implies a more resilient immune response, which may help centenarians cope better (Stanley et al., 2024).This cross-immunity hypothesis is particularly relevant in the context of elderly individuals who may have developed an immune memory toward viral strains “stored” over time. In Italy, centenarians born around the last mortality peaks of the Russian influenza pandemic may have benefited from a lasting immunity protection against SARS-CoV-2-like viral strains acquired in childhood.

Although further studies are needed, this theory suggests that some “Russian Flu” survivors developed lasting protective immunity, which may have been engaged by SARS-CoV-2.

Possible explanations for the resilience of centenarians to COVID-19 almost converge on a possible prior immunity, even if the possible agent responsible is still unknown (Poulain et al., 2021; Caruso et al., 2023; Lio et al., 2021; Guerini et al., 2021).

A Belgian research study found that, in 2020, centenarians born after August 1, 1918 in Belgium had a higher mortality rate than older centenarians (Poulain et al., 2021). A similar study was also conducted in Sicily (Italy) by Caruso et al. (2023) to calculate the crude excess mortality between 2019 and 2020 for centenarians born after 1918 and those born before 1919. Centenarians aged 100 or 101 in 2020 experienced a 61% higher mortality rate than the others. However, centenarians born before 1919 who were aged 102 years or older did not have a higher mortality rate (Caruso et al., 2023). The two studies used demographic data released by the relevant demographic organizations.

This body of research is part of a larger context of inquiry raised by the COVID-19 pandemic, which has stimulated interest in investigating acquired immunity and cellular memory as protective factors against emerging viral infections. The proposed research has several drawbacks. Initially, the total mortality rate, encompassing all deaths caused by COVID-19, is considered, as in the pandemic’s initial phase, certain cases are likely not being attributed to COVID-19. It is also possible that susceptibility to COVID-19 among the oldest is significantly affected by their specific living circumstances, particularly because many centenarians live in nursing homes. Moreover, these individuals were already among the most vulnerable and were likely less exposed to SARS-CoV-2 due to social isolation and protective measures implemented at the beginning of the pandemic. Apart from the preventive measures taken during the COVID-19 pandemic and the hypothesized prior immunity, the resilience of centenarians should be considered from a broader perspective, since genetic factors associated with a more efficient immune response and adaptive immunosenescence could also be involved.

We refrain from conducting direct blood tests on the centenarians, which leaves our theory regarding protection from cross-reactive immunity purely speculative. This study does not include new serological analyses to assess the presence of neutralizing antibodies or tests demonstrating cellular immune responses (T cells specific to SARS-CoV-2 and HCoV-OC43). However, the results of this manuscript also include previous investigations carried out at the IRCCS Policlinico San Matteo Foundation (Cassaniti et al., 2021). Lastly, the correlation between prior exposure to HCoV-OC43 and the resilience of the centenarians does not prove that such exposure was responsible for their increased resistance to SARS-CoV-2.

The current findings open up questions not only about the history of the” Russian Flu” but also about the immune resilience of entire generations exposed to past pandemics, showing how epidemiological history can offer valuable insights for dealing with future public health emergencies.

## Data Availability

The datasets presented in this study can be found in online repositories. The names of the repository/repositories and accession number(s) can be found in the article/Supplementary material.
