# Supplementary figures and images for: Phylogenetic and epidemiological insights into centenarians’ resilience to COVID-19: exploring the role of past coronavirus pandemics
Source: Front Microbiol. 2025 Apr 17;16:1572763. doi: 10.3389/fmicb.2025.1572763 (PMC12043687; doi:10.3389/fmicb.2025.1572763)

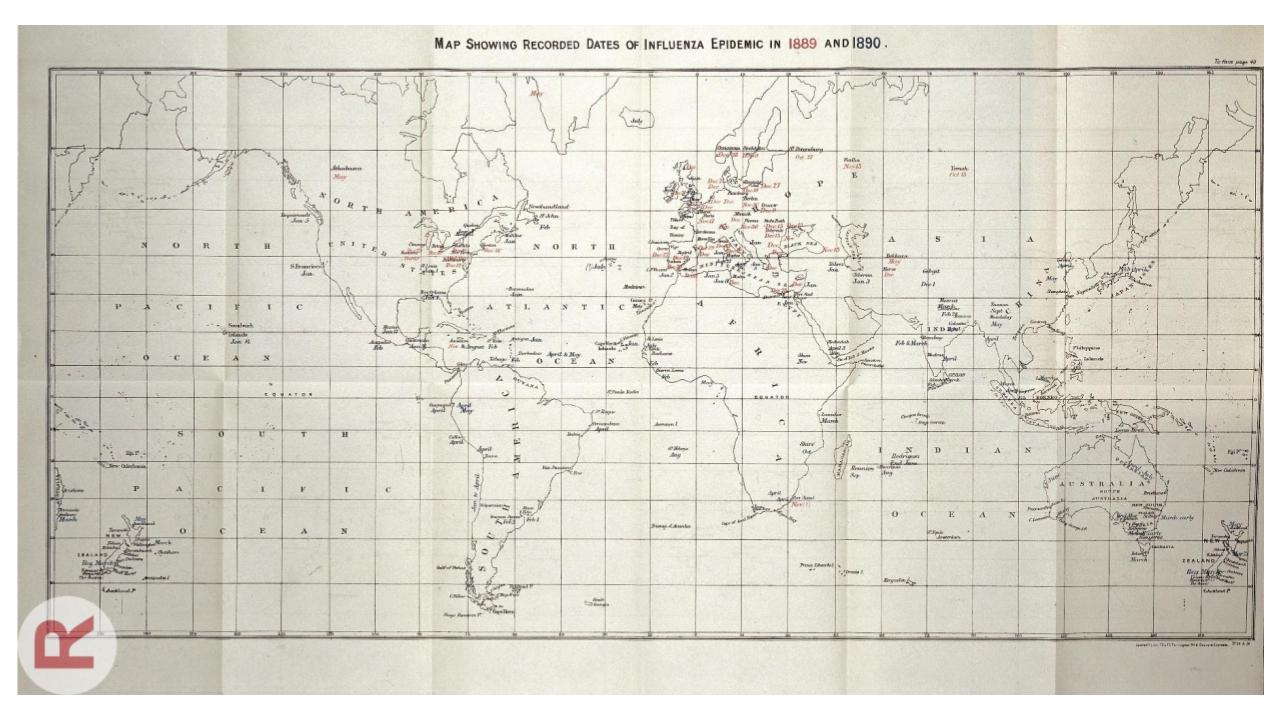

Supplement: Supplementary file 1 [file Image_1.jpeg]
